# Which is the most prominent spinous process in the cervico-thoracic spinal junction? A radiological study in a Mediterranean population sample

**DOI:** 10.1186/1748-7161-8-S2-O40

**Published:** 2013-09-18

**Authors:** Theodoros Grivas, Gerasimos Tsilimidos, Christos Verras, Konstantinos Botsios, Marianna Chatzisaroglou

**Affiliations:** 1 Department of Trauma and Orthopaedics, "Tzaneio" General Hospital, Piraeus, Greece

## Background

The spinous process (SP) of the seventh cervical vertebra (C7) is characterized as the most prominent, which makes it an anatomical landmark for the recognition of other SPs. However, is the C7 SP always longer and more prominent, or is there a deviation from this morphology? 

## Purpose

The purpose of this study was to answer the question above. 

## Methods

A total of 195 subjects were included in this study: 93 men (47.7%), with a mean age of 42.14 years, and 102 women (52.3%), with a mean age of 49.43. The length of the SP was determined to be the middle point of the SP’s origin, from the vertebral lamina up to the middle of its tip. The comparisons of continuous variables were done by the t-test, using the SPSS 20.0 package. 

## Results

In males, C7 SP is longer in 72.90% and T1 is longer in 27.1% (p <0,001); in females, C7 is longer in 42.7% and T1 is longer in 57.3% (p <0,001), respectively. Therefore, there is a statistically significant difference of C7 – T1 SP morphology. In males, the average length of C7 SP was 44,11 mm (range 23,70 mm to 61,90 mm); in females, the average length of C7 SP was 37,82 mm (range 26,10 mm to 54,20 mm). In males, the T1 SP length was 47,93 mm (range 24,20 mm to 58,70 mm); in females, the T1 SP length was 40,44 mm (ranging 24,40 mm to 53,10 mm). 

## Conclusions and discussion

The anatomical differences of C7 and T1 SP lengths are now described qualitatively and quantitatively in a Mediterranean population sample. It is found that there is a sexual dimorphism, with C7 SP in the males and T1 in females often being longer. The morphological patterns found in this study vary from those published earlier about scoliotic subjects, a finding that may implicate SP morphology in idiopathic scoliosis aetiology. Based on these results, the necessity of finding the correct anatomical landmark on the surface of the torso (SPs of C7 or T1) is confirmed for the purpose of clinical examination and for surface topography apparatus to correctly function in a scoliosis clinic. In addition, finding the correct anatomical landmark on the torso is essential to a number of other medical specialties using surface anatomical landmarks (e.g., general orthopedics, anaesthesiology and forensic medicine). 
